# Shrinkage and Warpage Minimization of Glass-Fiber-Reinforced Polyamide 6 Parts by Microcellular Foam Injection Molding

**DOI:** 10.3390/polym12040889

**Published:** 2020-04-11

**Authors:** Youngjae Ryu, Joo Seong Sohn, Chang-Seok Yun, Sung Woon Cha

**Affiliations:** Department of Mechanical Engineering, Yonsei University, 50, Yonsei-ro, Seodaemun-gu, Seoul 03722, Korea; yjryu1027@yonsei.ac.kr (Y.R.); ssamjjang87@yonsei.ac.kr (J.S.S.); changseok2614@yonsei.ac.kr (C.-S.Y.)

**Keywords:** microcellular foam injection molding, polyamide 6, glass fiber, shrinkage, warpage

## Abstract

Shrinkage and warpage of injection-molded parts can be minimized by applying microcellular foaming technology to the injection molding process. However, unlike the conventional injection molding process, the optimal conditions of the microcellular foam injection molding process are elusive because of core differences such as gas injection. Therefore, this study aims to derive process conditions to minimize the shrinkage and warpage of microcellular foam injection-molded parts made of glass fiber reinforced polyamide 6 (PA6/GF). Process factors and levels were first determined, with experiments planned accordingly. We simulated designed experiments using injection molding analysis software, and the results were analyzed using the Taguchi method, analysis of variance (ANOVA), and response surface methodology (RSM), with the ANOVA analysis being ultimately demonstrating the influence of the factors. We derived and verified the optimal combination of process factors and levels for minimizing both shrinkage and warpage using the Taguchi method and RSM. In addition, the mechanical properties and cell morphology of PA6/GF, which change with microcellular foam injection molding, were confirmed.

## 1. Introduction

The automotive industry is attempting to reduce automobile weight by replacing metal parts with plastic ones to improve gas mileage [1]. Injection molding is a suitable process for manufacturing automobile plastic parts with complex shapes [2]. This process consists of plastic resin pellets placed in an injection molding machine’s hopper, followed by entering a heated barrel containing the rotating screw to be melted. The melted resin is then transferred to the barrel’s front by rotation of the screw. Finally, the resin is injected into a mold of the desired shape and, after cooling, taken out as the solidified product [3].

There have been many studies on manufacturing plastic automobile parts using injection molding mainly using polypropylene (PP) and polyamide 6 (PA6)-based materials [4,5,6,7,8,9,10]. Whiteside et al. predicted the fiber orientation distribution of a clutch pedal made with glass fiber reinforced PA6 by using simulation software and confirmed that the distribution matched the experimental results [4]. Panthapulakkal et al. proposed the applicability of hemp/glass fiber reinforced PP composites for automotive parts by determining mechanical, thermal, and water absorption properties of the composites [6]. Park et al. minimized the weight and post-injection deformation of front body structure parts made of PA6 reinforced with 30 wt % short glass fiber [10].

Furthermore, the microcellular foaming process has been proposed to reduce the weight of the injection-molded product [11,12,13]. Microcellular foamed plastics are known to not only reduce the weight of any products compared to unfoamed products, but also to reduce shrinkage and warpage [14,15,16,17]. Kramschuster et al. measured the shrinkage and warpage of box-shaped PP parts using conventional and microcellular foam injection molding. In the case of microcellular foam injection molding, shrinkage, and warpage of the parts were reduced when compared with the conventional injection molding process [14]. Zafar et al. confirmed that shrinkage of ASTM standard rod-shaped parts gradually decreased as the weight of the samples decreased by microcellular foam injection molding. Glass-fiber-filled and unfilled acetal copolymers were used as the materials in the experiment, and they found that the shrinkage of both types reduces with increasing weight reduction [15]. Kim et al. studied the shrinkage and warpage of plate-shaped glass fiber-reinforced PP parts by using the microcellular foam injection molding process. When using this process, both shrinkage and warpage were reduced compared with unfoamed parts, especially in a direction perpendicular to the flow direction [16]. These advantages are useful to the automotive industry, where the dimensional stability of components is important.

The microcellular foam injection molding process starts with an injection of inert gas (e.g., N_2_) into the molten polymer resin, which is in a supercritical fluid state, and the gas and the resin subsequently form a single-phase in the form of a polymer-gas solution. As the solution is injected into the injection mold, a sudden pressure drop occurs, which causes the formation of microcells. Research on manufacturing automotive parts using this process is ongoing [18,19,20,21,22,23,24]. Kharbas et al. compared thermoplastic polyurethane foams made using supercritical gas-laden pellets with either a chemical blowing agent, a physical blowing agent, or co-physical blowing agents [22]. Comparison results showed that co-physical blowing agents lead to lower bulk density, better microstructure, and lower hysteresis loss ratios. Wang et al. confirmed the cell morphology and mechanical properties of the injection-molded microcellular foamed PP/talc composite and found that the addition of nano talc improved cell structure, strength, and stiffness and toughness when compared to pure PP foam [23]. Volpe et al. investigated the thermo-mechanical properties, density, and cell morphology of polyamide 66 (PA66) reinforced with glass fiber as a function of injection temperature, cavity thickness, and gas injection pressure [24]. In addition, analysis of variance (ANOVA) revealed that higher injection temperature, higher gas injection pressure, and increased thickness led to homogeneous foam and better mechanical properties.

Since gas is injected, unlike conventional injection molding processes, microcellular foam injection molding requires alternative processing conditions. Therefore, research to optimize these conditions is required. However, existing studies on the optimized microcellular foam injection molding process of PA6 for minimizing shrinkage and warpage are still insufficient.

In this study, microcellular foam injection molding process of glass fiber-reinforced PA6 was optimized to minimize the shrinkage and warpage of the injection-molded parts. Because PA6 is mainly used for automotive exterior parts, it must be optimized for the production of microcellular foamed parts. We predict that shrinkage and warpage depend on process factors considered to be important in microcellular foam injection molding process, such as melt and mold temperatures, packing and cooling times, and gas content. The experiments were designed considering each factor and level, and the results were analyzed using ANOVA, the Taguchi method, and response surface methodology (RSM).

## 2. Materials and Methods

### 2.1. Materials

PA6 reinforced with 18 wt% of glass fiber and 22 wt% of minerals was used as the test material. The polymer composite was manufactured by Kolon Plastics Inc. (KOPA6 KN133HBRR, Gyeongsang, Korea) and has the characteristics shown in Table 1.

### 2.2. Methods

**Figure 1:**
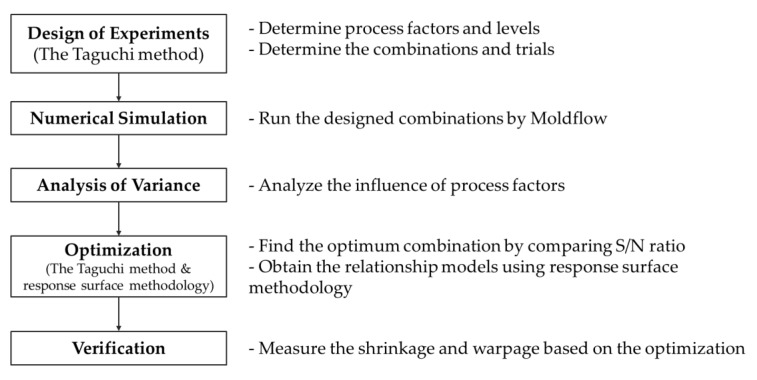
Flowchart of process optimization.

In order to achieve the purposed goal of this study, process optimization was carried out in several steps (Figure 1), using techniques such as design of experiments (DOE), ANOVA, the Taguchi method, and RSM.

#### 2.2.1. The Taguchi Method

The Taguchi method is a robust design method used for noise factors that cannot be controlled. Therefore, the concept of signal-to-noise ratio (S/N ratio) is introduced, and the condition where the S/N ratio is large is optimal. The equation for the S/N ratio in which the smaller value is a better characteristic is as follows [25]:

(1) $$\frac{Signal}{Noise}=\frac{S}{N}=-10log_{10}\overset{n}{\underset{i=1}{\sum }}\frac{y_{i}^{2}}{n}$$

where n is the number of data sets, and $y_{i}$ is the quantitative value of the data sets. In this study, n is the number of simulations, and $y_{i}$ corresponds to the value of shrinkage and warpage.

#### 2.2.2. Analysis of Variance

ANOVA compares the variance from each factor with the variance of the error, determining which factor is significant [26]. The influence of factors such as the degree of freedom (f), sum of squares (SS), and variance can be compared by calculating their contribution (%) as follows [27]:

(2) $$Degree\;of\;freedom\;for\;factor\;A=f_{A}=k_{A}-1$$

(3) $$Total\;degree\;of\;freedom=f_{t}=k_{t}-1$$

where $k_{A}$ is the number of results for factor A, and $k_{t}$ is the total number of results.

(4) $$SS_{A}=\frac{{\left(\sum^{\;}A_{1}\right)}^{2}}{k_{A}}+\frac{{\left(\sum^{\;}A_{2}\right)}^{2}}{k_{A}}+\frac{{\left(\sum^{\;}A_{3}\right)}^{2}}{k_{A}}-\frac{{\left(y_{1}+y_{2}+\cdots +y_{k_{t}}\right)}^{2}}{k_{t}}$$

(5) $$SS_{T}=\sum^{\;}(SS_{A}+SS_{B}+\cdots +SS_{E})$$

where SS is the sum of squares and $\sum^{\;}A_{i}$ is the sum of the $y_{i}$ when the level of factor A is ith.

(6) $$Variance\;for\;factor\;A=\frac{SS_{A}}{f_{A}}$$

(7) $$Contribution\;\left(\%\right)=\frac{SS_{A}}{SS_{T}}\times 100$$

#### 2.2.3. Response Surface Methodology

RSM analyzes the relationship several factors have with their result. Using RSM, we can determine the relationship between design factors and result values using the relationship equation and the response surface plot, ultimately finding the optimal response. The equation, called the response surface model, is expressed as a regression model as follows [28]:

(8) $$Y=a_{0}+\overset{k}{\underset{i=1}{\sum }}a_{i}x_{i}+\overset{k}{\underset{i=1}{\sum }}a_{ii}x_{i}x_{i}+\overset{k}{\underset{ij}{\sum }}a_{ij}x_{i}x_{j}$$

where Y is the response output (shrinkage and warpage) by the response surface model, and a corresponds to coefficients, x is level of factor, and k is the number of the factors.

#### 2.2.4. Experimental Setup

**Figure 2:**
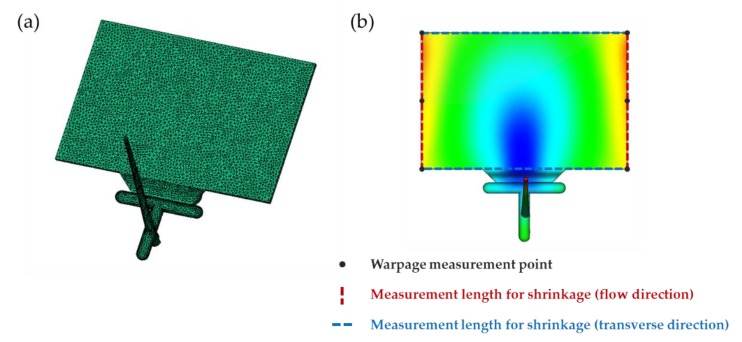
Shape of the test sample model: (**a**) mesh model, and (**b**) measurement points of shrinkage and warpage.

Simulations were performed using injection molding analysis software Autodesk Moldflow Insight (Framingham, The Commonwealth of Massachusetts, USA) to analyze the shrinkage and warpage of the injection-molded parts as a function of the process factors [29]. In order to compare the shrinkage and warpage tendency, we selected a thin rectangular shape as a test sample model for simulation, with a width, length, and thickness of 148, 98 mm, and 1.8 mm, respectively. The sample mesh in Moldflow consists of a total of 19,180 small triangles (Figure 2a). The generated mesh shows a match percentage of 94.6% compared with the shape of the sample model. Shrinkage in the flow direction (FD) was calculated based on the average value of the two lengths in the flow direction parallel to the flow of the resin (Figure 2b). Shrinkage in the transverse direction (TD) was calculated based on the average value of the two lengths in the transverse direction perpendicular to the flow direction (Figure 2b). Warpage was calculated as the average of three points on the left edge and three points on the right edge that showed the greatest warpage in the test sample model (Figure 2b).

Based on the factors expected to have an effect on shrinkage and warpage in microcellular foam injection molding process, we selected five factors and three levels (Table 2). The levels of melt temperature, mold temperature, and cooling time were selected as the recommended range for the material. The levels of gas content were selected by dividing the range (less than 1 wt %) mainly used in microcellular injection molding [30,31]. In this type of injection molding, the pressure and time of packing are set relatively small, because of the expanding gas in the melt resin [13]. Therefore, the packing pressure was fixed at 20% of the injection pressure, and the packing time was set between 0 and 2 s in this experiment. There are two types of microcellular foam injection molding processes: the full-shot process, in which the mold cavity is filled completely (similar as that for the conventional injection molding process), and the short-shot process, in which the mold cavity is partially filled. In the case of the short-shot process, the empty cavity is filled through the growth of the cells. In this study, the full-shot process was used for comparison with unfoamed conditions.

The experiment was designed using an orthogonal array table considering the selected factors and levels [32]. Table 3 shows a total of 27 designed experiments that were simulated by Moldflow. Next, the simulation results were analyzed by ANOVA, the Taguchi method, and RSM using the data analysis software Minitab in order to determine the optimal conditions for minimizing shrinkage and warpage of injection-molded parts [33].

#### 2.2.5. Experimental Verification

**Figure 3:**
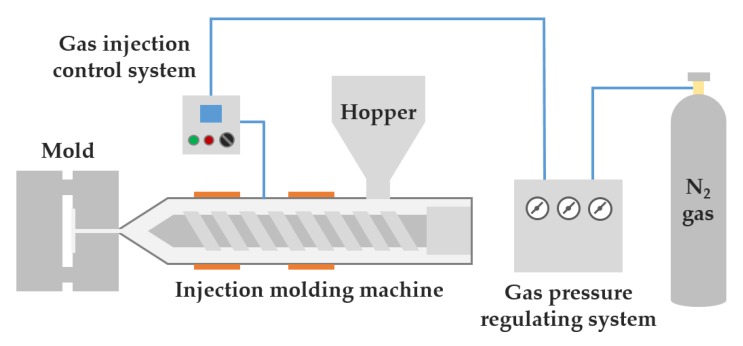
Schematic diagram of the microcellular foam injection molding process.

We injection-molded using a 120 tons clamping force injection molding machine (Woojin Plaimm Co., Ltd, Woojin Selex-E120, Chungcheong, Korea) to test the shrinkage and warpage of the unfoamed and microcellular foamed samples after drying the material (80 °C, 4 h). For the microcellular foam injection molding process, a gas injection port for injecting N_2_ gas (10 MPa) in a supercritical fluid state was connected to the barrel of the conventional injection molding machine (Figure 3).

Shrinkage and warpage of the test specimen were measured 48 h after injection molding and 10 samples were used in the measurement of each condition. The shrinkage and warpage values of each condition were calculated by excluding the maximum and minimum values and averaging over the remaining eight values. The shrinkage was derived using the following equation.

(9) $$Shrinkage\;\left(\%\right)=\frac{l_{mold}-l_{part}}{l_{mold}}\times 100$$

where $l_{mold}$ represents the length of the mold, and $l_{part}$ represents the length of the injection-molded parts. When the sample was placed on the floor, we measured the maximum height (mm) of the bending as warpage.

#### 2.2.6. Mechanical Properties

In order to compare the mechanical properties of unfoamed and microcellular foamed parts, test specimens of tensile strength, flexural strength, and impact strength were injection-molded. The mechanical properties of ten test samples for each condition were measured, and the average strength was calculated, excluding the maximum and minimum values. We used a universal testing machine (QMESYS Co., Ltd., QM-100T, Gyeonggi, Korea) to measure the tensile strength and flexural modulus, and an Izod impact tester (Salt Co., Ltd., ST-120, Incheon, Korea) to measure impact strength. Mechanical properties were measured by applying methods specified by the American Society for Testing and Materials (ASTM). Tensile strength was derived by tensioning test specimens of type 1 (165 mm × 19 mm × 3.3 mm) as per the ASTM D638 method. Flexural modulus and impact strength were measured with the ASTM D790 and ASTM D256 methods, respectively.

#### 2.2.7. Cell Morphology

A scanning electron microscope (SEM) (JEOL Ltd, JEOL-7001F, Tokyo, Japan) was used to observe the cell morphology by enlarging the cross-sections of the unfoamed and microcellular foamed specimens. The average cell diameter was calculated by averaging the diameters of all cells in the SEM image, and the cell diameters were measured using ImageJ, an image analysis software. The foaming ratio is calculated by the following equations from the density of the unfoamed sample and the density of the foamed sample.

(10) $$Foaming\;ratio\;\left(\%\right)=\frac{\rho_{unfoamed}-\rho_{foamed}}{\rho_{unfoamed}}\times 100$$

where $\rho_{unfoamed}$ represents the density of the unfoamed parts, and $\rho_{foamed}$ represents the density of the foamed parts.

## 3. Results and Discussion

### 3.1. The Taguchi Method

**Figure 4:**
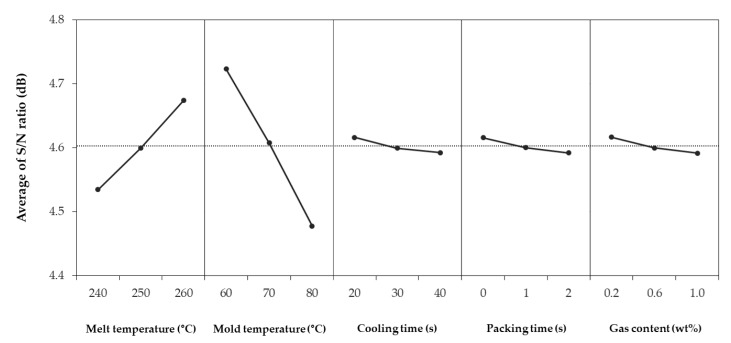
Average of S/N ratio for shrinkage in the flow direction.

**Figure 5:**
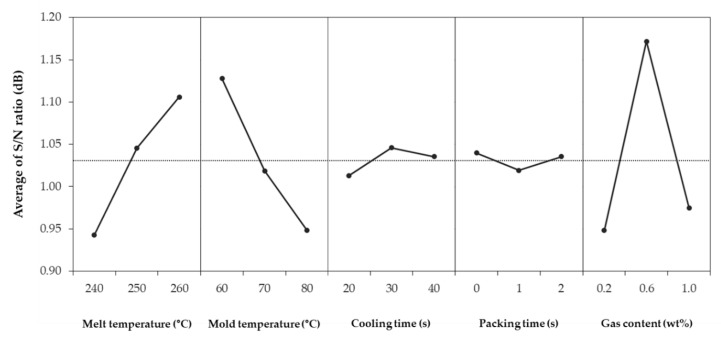
Average of S/N ratio for shrinkage in the transverse direction.

**Figure 6:**
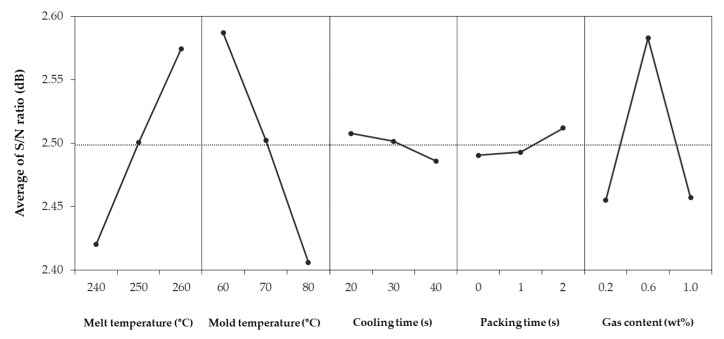
Average of S/N ratio for warpage.

Table 4 shows shrinkage in the FD, shrinkage in the TD, warpage, and S/N ratios for each combination. The 27 simulations showed an average shrinkage in the FD of 0.589%, average shrinkage in the TD of 0.888%, and warpage of 0.7501 mm. The difference of the S/N ratio between each factor and level is shown in Figure 4, Figure 5, Figure 6. The larger the S/N ratio, the more optimal the level.

The optimum combination for obtaining the smallest shrinkage in the FD is a melt temperature of 260 °C, mold temperature of 60 °C, cooling time of 20 s, packing time of 0 s, and gas content of 0.2 wt % (Figure 4). The optimum combination for obtaining the smallest shrinkage in the TD is a melt temperature of 260 °C, mold temperature of 60 °C, cooling time of 30 s, packing time of 0 s, and gas content of 0.6 wt % (Figure 5). In contrast, the optimum combination for the smallest warpage is a melt temperature of 260 °C, mold temperature of 60 °C, cooling time of 20 s, packing time of 2 s, and gas content of 0.6 wt % (Figure 6).

Both, shrinkage and warpage showed the most optimal results at a melt temperature of 260 °C and mold temperature of 60 °C. This tendency is similar to the conventional injection molding in which the shrinkage is smaller as melt temperature increase, and as mold temperature decrease [34].

However, the minimum shrinkage conditions were different from the warpage conditions in terms of the cooling time, packing time, and gas content. There were existing studies that the optimum condition for the shrinkage and that for the warpage is different [35]. First, the shrinkage and warpage did not decrease further, as the cooling time continued to increase. This is because the longer the cooling time, the longer the cell growth time of the microcells. As the cell size increases, the uniformity of the overall cell size decreases, and the possibility of causing warpage increases. Also, as the injected gas takes the place of the packing pressure role, the packing time at which shrinkage and warpage is minimized does not show a certain trend. Lastly, the gas content tended to move away from the optimum condition when it exceeded a certain content. Previous studies show that the cell size increases with an increase of gas content [36]. As the cell size increases, the warpage also increases because of increased cell non-uniformity.

Table 5 shows the optimal combinations of shrinkage and warpage by the Taguchi method, where the average levels of both were used to minimize both shrinkage and warpage.

### 3.2. Analysis of Variance

In ANOVA, if the P-value is less than 0.05, the factor is considered statistically significant. For the shrinkage in the FD, melt and mold temperatures were considered significant factors (Table 6). Regarding significant factors, mold temperature had by far the biggest influence, followed by melt temperature. In contrast, cooling time, packing time, and gas content were not significant factors.

For the shrinkage in the TD, melt and mold temperatures and gas content were the significant factors (Table 7). The significance of these factors is in the order of gas content, mold temperature, and melt temperature. The contribution of gas content to the shrinkage in the TD was 49.19%, which was critical compared to other process factors. Here, it can be seen how large the effect of gas content on the shrinkage in comparison to other process factors in microcellular foam injection molding is. In particular, the effect of gas content on the shrinkage in the TD was larger than that on the shrinkage in the FD. This is because incorporated glass fiber in PA6/GF reduces shrinkage in the FD by alignment with the flow direction before foam affects the shrinkage in the FD. Therefore, the effect of foaming on the shrinkage in the TD is relatively larger than that on the shrinkage in the FD. Cooling time and packing time were not significant factors for shrinkage in the FD.

For warpage, melt temperature, mold temperature, and gas content were the significant factors, similar as that for shrinkage in the TD (Table 8). However, the significance of these factors shows a different order to the shrinkage in the TD. The significance of the factors is in the order of mold temperature, melt temperature, and gas content. Cooling time and packing time were not significant factors for warpage.

In previous studies, the influence of each factor was analyzed to change according to the properties of the material. Ozcelik et al. confirmed that mold and melt temperatures, packing pressure, and cooling time are all significant factors when simulating the warpage of injection-molded thin shell plastic parts made of polyoxymethylene (POM) [37]. In their study, the influence of packing and cooling times is the smallest among all factors, but these factors are still significant. In another study, melt temperature and cooling time were significant for shrinkage, but mold temperature was found to be insignificant when simulating the shrinkage of injection-molded DVD cover products made with acrylonitrile butadiene styrene (ABS) [38]. In Chen’s research, packing and cooling times were significant factors for the warpage of injection-molded polybutylene terephthalate (PBT) samples, but melt temperature was insignificant [39].

### 3.3. Response Surface Methodology

**Figure 7:**
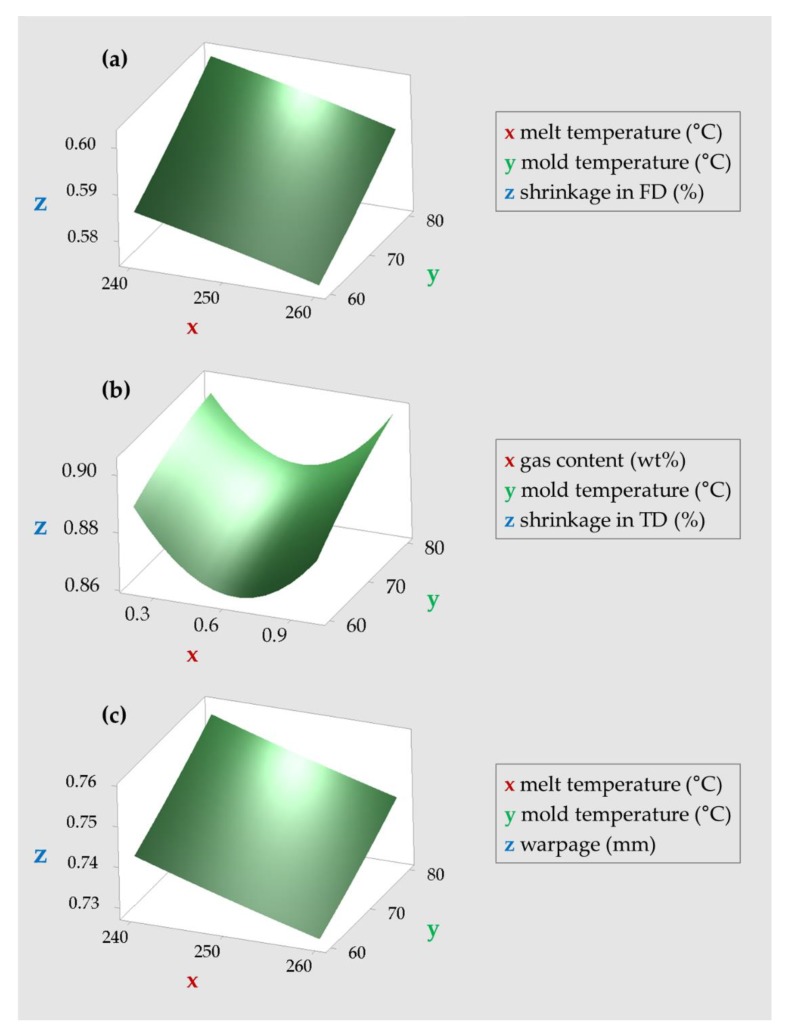
Surface plots of (**a**) shrinkage in the flow direction, (**b**) shrinkage in the transverse direction, and (**c**) warpage.

The relationship between the process factors and result values (shrinkage and warpage) were obtained by using RSM Equations (11), (12), and (13). The relationship between two process factors and response values is shown in Figure 7. We selected and plotted the two factors that had the greatest effect on each result. The shrinkage in the FD is minimized as melt and mold temperatures decrease, while the shrinkage in TD is minimized as the mold temperature decreases and gas content approaches 0.6 wt%. The warpage is minimized as the melt and mold temperatures decrease.

Table 9 shows the optimal combination derived using RSM. Optimal combinations are divided into minimizing only shrinkage, minimizing only warpage, and minimizing both.

(11) $$\begin{array}{ll} Y_{shrinkage\_FD}= & 0.483+0.00092x_{1}+0.00018x_{2}+0.000375x_{3}+0.00076x_{4}\\ & +0.0240x_{5}-0.000003x_{1}x_{1}+0.000006x_{2}x_{2}-0.000003x_{3}x_{3}\\ & -0.00028x_{4}x_{4}-0.00174x_{5}x_{5}-0.000208x_{2}x_{5}-0.000208x_{3}x_{5}\\ & +0.00104x_{4}x_{5} \end{array}$$

(12) $$\begin{array}{ll} Y_{shrinkage\_TD}= & 2.55-0.01251x_{1}+0.00320x_{2}-0.00138x_{3}+0.00604x_{4}-0.4431x_{5}\\ & +0.000022x_{1}x_{1}-0.000019x_{2}x_{2}+0.000022x_{3}x_{3}-0.00194x_{4}x_{4}\\ & +0.1337x_{5}x_{5}+0.000937x_{1}x_{5}+0.000729x_{2}x_{5}-0.000104x_{3}x_{5}\\ & -0.00313x_{4}x_{5} \end{array}$$

(13) $$\begin{array}{ll} Y_{warpage}= & 1.233-0.00267x_{1}-0.00022x_{2}-0.000187x_{3}+0.00112x_{4}-0.3055x_{5}\\ & +0.000003x_{1}x_{1}+0.000005x_{2}x_{2}+0.000004x_{3}x_{3}-0.00073x_{4}x_{4}\\ & +0.06821x_{5}x_{5}+0.000784x_{1}x_{5}+0.000390x_{2}x_{5}+0.000038x_{3}x_{5}\\ & -0.00092x_{4}x_{5} \end{array}$$

### 3.4. Optimization Comparison

Previously, the Taguchi method and RSM were used to derive the optimum combination of process factors and levels for minimizing shrinkage and warpage. Two additional simulations were performed to confirm the optimum combinations by the two methods (Table 10). The resulting combinations yielded values smaller than the average shrinkage and warpage of the 27 simulations (where the average shrinkage in the FD is 0.589%, and the average shrinkage in the TD is 0.888%, and the average warpage is 0.7501 mm). The Taguchi method minimized the shrinkage in the TD and warpage more than RSM. Therefore, the Taguchi method is more optimized for reducing the shrinkage and warpage than RSM.

### 3.5. Experimental Verification

When comparing the optimization methods previously, we found that a combination with the Taguchi method is the most optimal combination. Therefore, the simulation and experimental results were compared with the optimal combination by the Taguchi method. Table 11 shows the comparison of the simulation and experimental results. In the case of shrinkage, the experimental results were smaller than those of the simulation. The experimental shrinkage in the FD was approximately 0.189% smaller than that observed in the simulation, and the experimental shrinkage in the TD was approximately 0.252% smaller than that in the simulation. However, in the case of warpage, the simulation and experimental results were similar. The difference between the two was only 0.0061 mm. In the simulation, one constant cell size will occur by microcellular foaming, but various cell sizes occur in actual foaming. In addition, the amounts of gas dissolution and diffusion applied to the simulation may be different from the actual experiment as per experimental conditions. Therefore, we analyzed that errors occurred between the simulation and experimental values due to these points.

### 3.6. Effects of Microcellular Foaming

To confirm the effect of microcellular foaming, we compared shrinkage, warpage, mechanical properties, and SEM images of unfoamed and microcellular foamed PA6/GF samples under optimal conditions. The only difference in the process conditions between unfoamed and foamed samples was the gas content.

**Figure 8:**
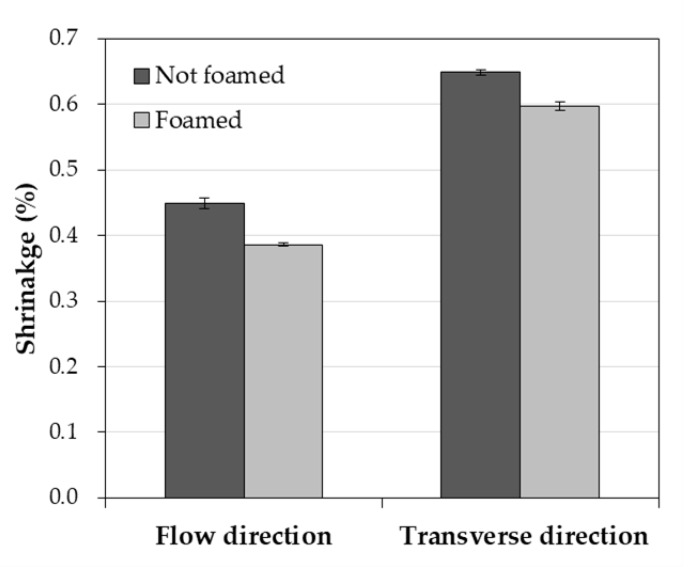
Measured shrinkages in the flow direction and the transverse direction.

First, the shrinkage in the FD, which was 0.449% when not foamed, decreased by approximately 0.063% to 0.386% after foaming (Figure 8). In addition, the shrinkage in the TD, which was 0.649% when not foamed, decreased by approximately 0.051% to 0.598% after foaming (Figure 8). Lastly, when not foamed, the warpage was 11.825 mm, but after foaming, it decreased by about 11.11 mm to 0.715 mm. There was a significant improvement effect on the reduction of warpage rather than the shrinkage. Therefore, microcellular foaming is expected to be a great help in improving the dimensional stability of the injection-molded PA6/GF parts.

**Figure 9:**
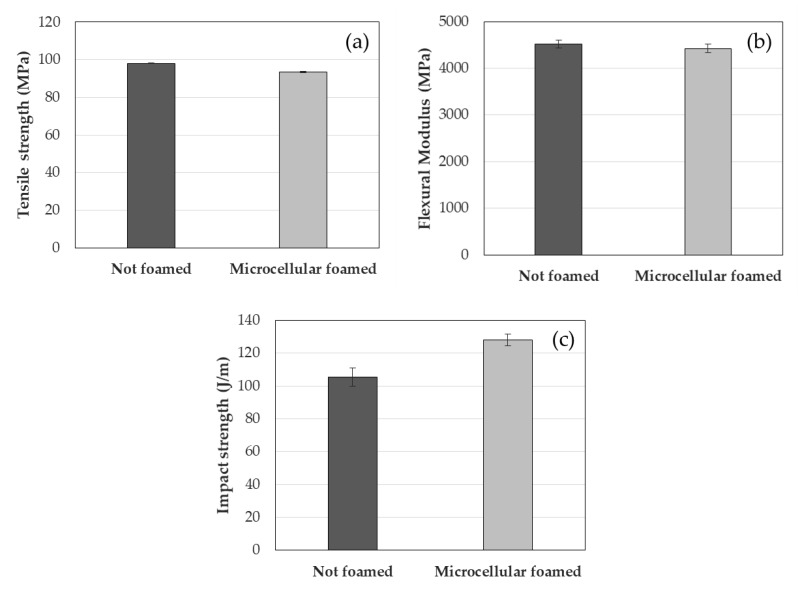
Mechanical properties of unfoamed and microcellular foamed PA6/GF: (**a**) tensile strength, (**b**) flexural modulus, and (**c**) impact strength.

The tensile strength of the PA6/GF sample decreased by about 4.6% from 98.0 to 93.5 MPa after foaming, and the flexural modulus decreased by about 2.1% from 4522 to 4427 MPa after foaming (Figure 9a,b). In contrast, the impact strength increased approximately 21.4% from 105.4 to 128.0 J/m after foaming (Figure 9c). Although the tensile strength and flexural modulus decreased slightly by foaming, the impact strength increased relatively, so it is expected that microcellular foaming would be useful to improve the impact strength of unfoamed parts. In the case of existing research, it was analyzed that the impact strength of injection-molded parts can be improved by using microcellular foam injection molding for brittle materials [40,41]. As PA6/GF is also a brittle material, it was analyzed that the impact strength is greatly improved by foaming.

**Figure 10:**
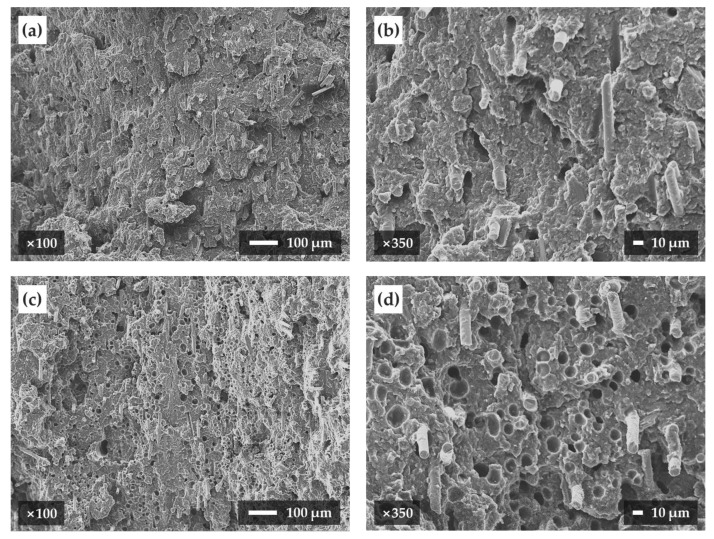
Cross-section images of samples taken by a scanning electron microscope: (**a**) unfoamed sample magnified 100 times, (**b**) unfoamed sample magnified 350 times, (**c**) foamed sample magnified 100 times, and (**d**) foamed sample magnified 350 times.

We observed enlarged images of the cross-sections of the unfoamed and microcellular foamed samples using SEM (Figure 10). First, in the case of the unfoamed sample, there were no cells in the cross-section and the glass fibers had a diameter of about 10 µm. However, in the case of the foamed sample, cells were visible in the cross-section of the foamed sample. Based on the SEM image of Figure 10d, the average cell diameter is 9.87 µm. Additionally, the average foaming ratio of the foamed samples is 8.39%.

## 4. Conclusions

In this study, process factors were selected with the objective of minimizing the shrinkage and warpage of PA6/GF in microcellular foam injection molding process. These factors were melt temperature, mold temperature, cooling time, packing time, and gas content. Additionally, three levels of each factor were determined according to the recommended level range.

Based on the selected factors and levels, DOE was used, and 27 simulations were performed through the injection molding analysis software Autodesk Moldflow Insight. The results were analyzed using the Taguchi method, ANOVA and RSM to determine the influence and optimal combination of process factors.

In case of the Taguchi method, both shrinkage and warpage were minimized when the melt temperature was 260 °C, mold temperature was 60 °C, cooling time was 23.33 s, packing time was 0.67 s, and gas content was 0.47 wt%. As a result of analyzing the influence of each factor by ANOVA, it was observed that the mold and melt temperatures had a significant effect on shrinkage and warpage overall; except in the case of the shrinkage in the FD, gas content was also significant. For the microcellular foam injection molding process, it was found that the influence of gas content is as important as the existing injection molding process factors. In contrast, cooling and packing times were determined to be relatively insignificant. For RSM, shrinkage and warpage were minimized at a melt temperature of 260 °C, a mold temperature of 60 °C, a cooling time of 26.26 s, a packing time of 0 s, and a gas content of 0.56 wt%.

When comparing the shrinkage and warpage under the two conditions derived from the Taguchi method and RSM, the combination derived from the Taguchi method showed smaller shrinkage and warpage in the simulation. In conclusion, the combination from the Taguchi method was the optimum condition in comparison with RSM. The measured shrinkage and warpage of the optimal combination are as follows: the shrinkage in the FD was 0.386%, the shrinkage in the TD was 0.598%, and the warpage was 0.715 mm.

Additionally, the unfoamed and microcellular foamed samples were compared experimentally to confirm shrinkage, warpage, mechanical properties, and cell morphology. The foamed samples exhibited reduced shrinkage and warpage relative to the unfoamed samples, and an improved impact strength. Lastly, it was confirmed that microcells having on average size of 9.87 µm were formed in the cross-section of the foamed sample.

## Figures and Tables

**Table 1:** Material properties.

Property	Value
Melt density (g/cm^3^)	1.3024
Solid density (g/cm^3^)	1.4824
Elastic modulus, 1st principal direction (MPa)	8529
Elastic modulus, 2nd principal direction (MPa)	5572
Shear modulus (MPa)	2215
Recommended melt temperature range (°C)	240–260

**Table 2:** Selected factors and levels.

Level	Factor				
	Melt Temperature (°C)	Mold Temperature (°C)	Cooling Time (s)	Packing Time (s)	Gas Content (wt %)
	A	B	C	D	E
1	240	60	20	0	0.2
2	250	70	30	1	0.6
3	260	80	40	2	1.0

**Table 3:** Design of experiments.

No.	A	B	C	D	E
1	240	60	20	0	0.2
2	240	60	20	0	0.6
3	240	60	20	0	1.0
4	240	70	30	1	0.2
5	240	70	30	1	0.6
6	240	70	30	1	1.0
7	240	80	40	2	0.2
8	240	80	40	2	0.6
9	240	80	40	2	1.0
10	250	60	30	2	0.2
11	250	60	30	2	0.6
12	250	60	30	2	1.0
13	250	70	40	0	0.2
14	250	70	40	0	0.6
15	250	70	40	0	1.0
16	250	80	20	1	0.2
17	250	80	20	1	0.6
18	250	80	20	1	1.0
19	260	60	40	1	0.2
20	260	60	40	1	0.6
21	260	60	40	1	1.0
22	260	70	20	2	0.2
23	260	70	20	2	0.6
24	260	70	20	2	1.0
25	260	80	30	0	0.2
26	260	80	30	0	0.6
27	260	80	30	0	1.0

**Table 4:** Experimental results and S/N ratios.

No.	Shrinkage in the Flow Direction (%)	S/N Ratio	Shrinkage in the Transverse Direction (%)	S/N Ratio	Warpage (mm)	S/N Ratio
1	0.580	4.73144	0.900	0.91515	0.7550	2.44068
2	0.585	4.65688	0.875	1.15984	0.7446	2.56173
3	0.585	4.65688	0.890	1.01220	0.7474	2.52874
4	0.590	4.58296	0.915	0.77158	0.7635	2.34382
5	0.595	4.50966	0.885	1.06113	0.7516	2.48007
6	0.595	4.50966	0.895	0.96354	0.7549	2.44260
7	0.605	4.36489	0.915	0.77158	0.7704	2.26567
8	0.600	4.43697	0.890	1.01220	0.7559	2.43052
9	0.605	4.36489	0.910	0.81917	0.7682	2.29089
10	0.580	4.73144	0.885	1.06113	0.7455	2.55085
11	0.580	4.73144	0.860	1.31003	0.7316	2.71433
12	0.585	4.65688	0.880	1.11035	0.7454	2.55241
13	0.590	4.58296	0.890	1.01220	0.7550	2.44144
14	0.590	4.58296	0.875	1.15984	0.7435	2.57496
15	0.585	4.65688	0.895	0.96354	0.7554	2.43646
16	0.600	4.43697	0.905	0.86703	0.7620	2.36109
17	0.595	4.50966	0.885	1.06113	0.7511	2.48566
18	0.595	4.50966	0.905	0.86703	0.7595	2.38906
19	0.575	4.80664	0.885	1.06113	0.7424	2.58763
20	0.580	4.73144	0.855	1.36068	0.7288	2.74783
21	0.575	4.80664	0.875	1.15984	0.7414	2.59914
22	0.580	4.73144	0.890	1.01220	0.7427	2.58354
23	0.585	4.65688	0.865	1.25968	0.7329	2.69970
24	0.585	4.65688	0.895	0.96354	0.7482	2.52003
25	0.590	4.58296	0.885	1.06113	0.7478	2.52390
26	0.590	4.58296	0.875	1.15984	0.7454	2.55241
27	0.595	4.50966	0.900	0.91515	0.7625	2.35520

**Table 5:** Optimum condition defined by the Taguchi method.

Optimization	A	B	C	D	E
Shrinkage in the flow direction	260	60	20	0	0.2
Shrinkage in the transverse direction	260	60	30	0	0.6
Warpage	260	60	20	2	0.6
Shrinkage and warpage	260	60	23.33	0.67	0.47

**Table 6:** Analysis of variance for shrinkage in the flow direction.

Design Parameters	Degree of Freedom	Sum of Squares	Mean Squares	F-Ratio (%)	P-Value	Contribution (%)
A	2	0.087106	0.043553	23.40	0.000	23.72
B	2	0.271923	0.135961	73.06	0.000	74.07
C	2	0.002643	0.001321	0.71	0.506	0.72
D	2	0.002581	0.001291	0.69	0.514	0.70
E	2	0.002904	0.001452	0.78	0.475	0.79
Error	16	0.029777	0.001861			
Total	26	0.396933				

**Table 7:** Analysis of variance for shrinkage in the transverse direction.

Design Parameters	Degree of Freedom	Sum of Squares	Mean Squares	F-Ratio (%)	P-Value	Contribution (%)
A	2	0.122273	0.061136	21.10	0.000	22.43
B	2	0.147367	0.073683	25.44	0.000	27.05
C	2	0.005089	0.002545	0.88	0.435	0.94
D	2	0.002133	0.001066	0.37	0.698	0.39
E	2	0.268008	0.134004	46.26	0.000	49.19
Error	16	0.046349	0.002897			
Total	26	0.591218				

**Table 8:** Analysis of variance for warpage.

Design Parameters	Degree of Freedom	Sum of Squares	Mean Squares	F-Ratio (%)	P-Value	Contribution (%)
A	2	0.106579	0.053289	27.55	0.000	29.99
B	2	0.147627	0.073814	38.16	0.000	41.55
C	2	0.002265	0.001133	0.59	0.568	0.64
D	2	0.002471	0.001236	0.64	0.541	0.70
E	2	0.096387	0.048194	24.91	0.000	27.12
Error	16	0.030951	0.001934			
Total	26	0.386281				

**Table 9:** Optimum condition by response surface methodology.

Optimization	A	B	C	D	E
Shrinkage in the flow direction	260	60	20	0	0.2
Shrinkage in the transverse direction	260	60	32.53	0	0.6
Warpage	260	60	20	2	0.58
Shrinkage & warpage	260	60	26.26	0	0.56

**Table 10:** Comparison of optimum combinations.

Method	A	B	C	D	E	Shrinkage in the Flow Direction (%)	Shrinkage in the Transverse Direction (%)	Warpage (mm)
The Taguchi method	260	60	23.33	0.67	0.47	0.575	0.850	0.7211
Response surface methodology	260	60	26.26	0	0.56	0.575	0.855	0.7281

**Table 11:** Comparison of simulation and experimental results.

	Shrinkage in the Flow Direction (%)	Shrinkage in the Transverse Direction (%)	Warpage (mm)
Simulation	0.575	0.850	0.7211
Experiment	0.386	0.598	0.715
